# A 3D mixed reality visualization of network topology and activity results in better dyadic cyber team communication and cyber situational awareness

**DOI:** 10.3389/fdata.2023.1042783

**Published:** 2023-01-27

**Authors:** Torvald F. Ask, Kaur Kullman, Stefan Sütterlin, Benjamin J. Knox, Don Engel, Ricardo G. Lugo

**Affiliations:** ^1^Department of Information Security and Communication Technology, Norwegian University of Science and Technology, Gjøvik, Norway; ^2^Faculty of Health, Welfare and Organization, Østfold University College, Halden, Norway; ^3^Doctoral School of Information and Communication Technology, Institute of Computer Science, Tallinn University of Technology, Tallinn, Estonia; ^4^Center for Space Sciences and Technology, University of Maryland, Baltimore County, Baltimore, MD, United States; ^5^Faculty of Computer Science, Albstadt-Sigmaringen University, Sigmaringen, Germany; ^6^Centre for Digital Forensics and Cybersecurity, Tallinn University of Technology, Tallinn, Estonia; ^7^Norwegian Armed Forces Cyber Defense, Oppland, Norway

**Keywords:** mixed reality, 3D network topology visualization, cyber team communication, Virtual Data Explorer, shared mental model, cyber situational awareness, human factors, cybersecurity

## Abstract

**Background:**

Cyber defense decision-making during cyber threat situations is based on human-to-human communication aiming to establish a shared cyber situational awareness. Previous studies suggested that communication inefficiencies were among the biggest problems facing security operation center teams. There is a need for tools that allow for more efficient communication of cyber threat information between individuals both in education and during cyber threat situations.

**Methods:**

In the present study, we compared how the visual representation of network topology and traffic in 3D mixed reality vs. 2D affected team performance in a sample of cyber cadets (*N* = 22) cooperating in dyads. Performance outcomes included network topology recognition, cyber situational awareness, confidence in judgements, experienced communication demands, observed verbal communication, and forced choice decision-making. The study utilized network data from the NATO CCDCOE 2022 Locked Shields cyber defense exercise.

**Results:**

We found that participants using the 3D mixed reality visualization had better cyber situational awareness than participants in the 2D group. The 3D mixed reality group was generally more confident in their judgments except when performing worse than the 2D group on the topology recognition task (which favored the 2D condition). Participants in the 3D mixed reality group experienced less communication demands, and performed more verbal communication aimed at establishing a shared mental model and less communications discussing task resolution. Better communication was associated with better cyber situational awareness. There were no differences in decision-making between the groups. This could be due to cohort effects such as formal training or the modest sample size.

**Conclusion:**

This is the first study comparing the effect of 3D mixed reality and 2D visualizations of network topology on dyadic cyber team communication and cyber situational awareness. Using 3D mixed reality visualizations resulted in better cyber situational awareness and team communication. The experiment should be repeated in a larger and more diverse sample to determine its potential effect on decision-making.

## 1. Introduction

Decision-making in Cyber Threat Situations (CTSs) is subject to many challenges due to the interconnectedness between decision-making agents and assets in cyber and physical space, and the high levels of uncertainty inherent to the cyber domain (Jøsok et al., 2016). This results in decision-making often having to be made on an insufficient information basis which makes it difficult to predict the impact of decisions on own and third-party assets, as well as on adversarial behavior (Jøsok et al., 2016). Other challenges to decision-making include competence differences between analyst-level and decision-making personnel (Knox et al., 2018), which are roles that often are assigned to different individuals within organizations doing cybersecurity operations (e.g., Security Operation Centers; SOCs).

**Figure 1:**
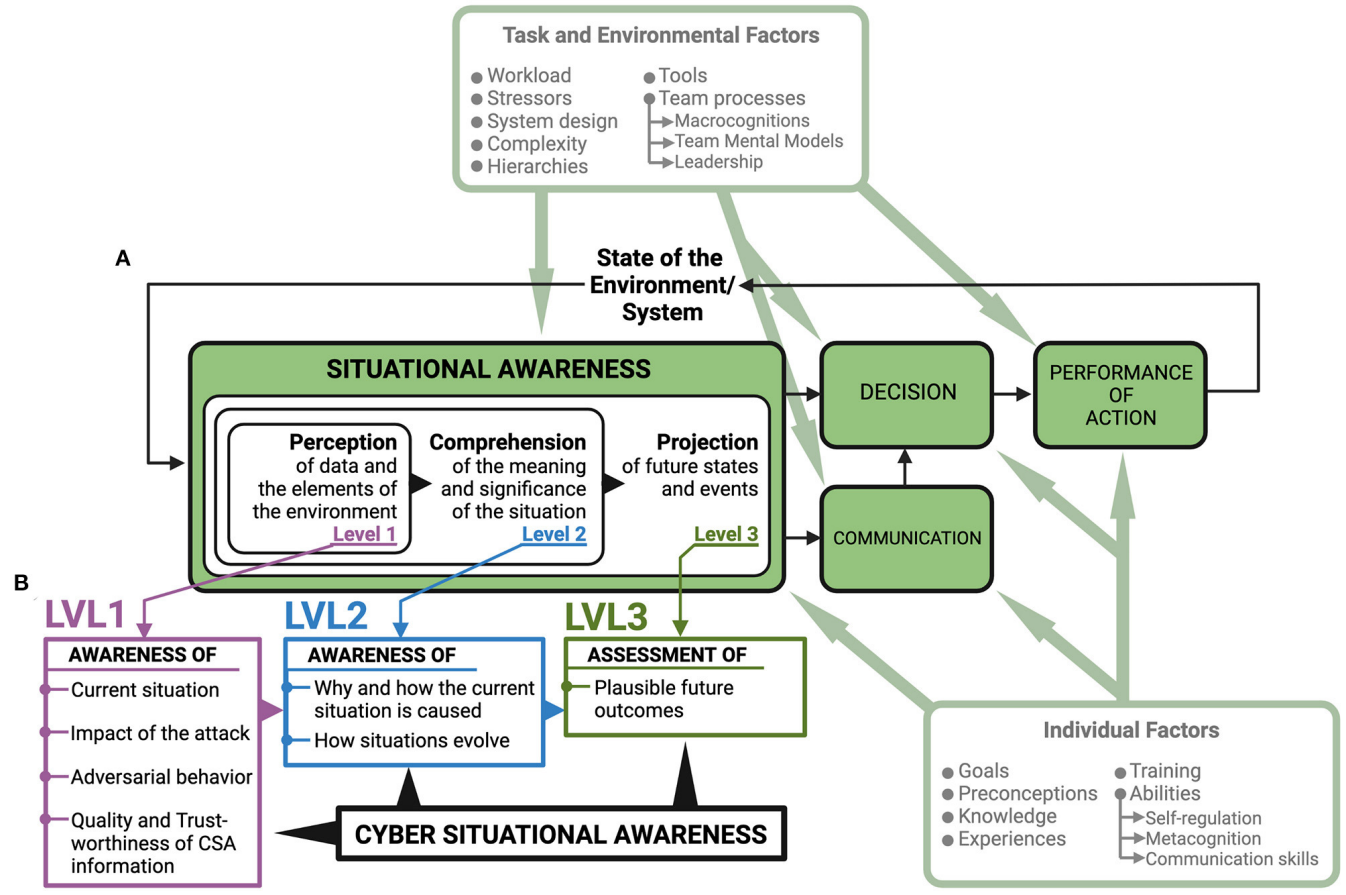
Situational Awareness model with suggested requirements for achieving Cyber Situational Awareness. **(A)** Situational Awareness is achieved in three stages (Endsley, 1995). To account for the separation between analysts and decision-makers in SOCs (Knox et al., 2018; Ask et al., 2021a), a “communication” element has been added to the model. **(B)** Seven requirements that can be organized under the Endsley model need to be met to achieve Cyber Situational Awareness for cyber defense (Barford et al., 2009). Establishing Cyber Situational Awareness, communicating for shared Cyber Situational Awareness, and decision-making based on Cyber Situational Awareness is influenced by individual factors such as emotion, metacognition, self-regulation, and communication skills (Jøsok et al., 2016, 2019; Knox et al., 2017, 2018; Ask et al., 2021b, 2023; Sütterlin et al., 2022) and task and environmental factors such as team-processes including macrocognitions, team mental models, and leadership (Jøsok et al., 2017; Ask et al., 2021a). Modified from Lankton (2007).

Due to the interconnectedness between assets and decision-making agents in the cyber and physical domains and the resulting human-human and human-machine interactions, cybersecurity operations unfold in a complex sociotechnical system. According to the Situational Awareness (SA) model (Figure 1A) proposed by Endsley (1988, 1995), establishing SA for decision-making in sociotechnical systems is achieved in three levels, where all levels must be achieved in order to have full SA.

SA Level 1 is the perception stage and involves perceiving the elements in a situation. SA Level 2 is the comprehension stage and involves understanding the relationship between the perceived elements. SA Level 3 involves using the understanding of the relationship between the elements to predict future states of the system that the situation is occurring in, and how those future states will be affected by decision-making (Endsley, 1995).

In a cybersecurity setting, SA is increasingly referred to as Cyber SA (CSA; Barford et al., 2009; Franke and Brynielsson, 2014). Extending on the formal definition of SA (Endsley, 1988), CSA is considered a subset of SA and can in general terms be defined as “*the perception of the elements in the [cyber] environment within a volume of time and space, the comprehension of their meaning and the projection of their status in the near future*” (Franke and Brynielsson, 2014, p. 4). It should be noted, however, that it is acknowledged that actions in the physical domain may influence events in cyberspace and *vice versa* (Jøsok et al., 2016). Consequently, stakeholders and decision-makers are often required to have a SA that simultaneously accounts for the impact of decisions in both the cyber and the physical domain.

Seven requirements for achieving CSA for cyber defense decision-making have been suggested (Barford et al., 2009). These requirements can be arranged under the SA model proposed by Endsley (Figure 1B). To achieve SA Level 1 during a CTS, one must have perceived indicators of compromise allowing for (1) awareness of the current situation; (2) awareness of the impact of the attack; (3) awareness of adversarial behavior; and (4) awareness of the quality and trustworthiness of CSA information. To achieve SA Level 2, one must have (5) awareness of why and how the current situation is caused (e.g., if it is an automatic or directed attack), and (6) awareness of how situations evolve. To achieve SA Level 3, one must be able to (7) assess plausible future outcomes.

Decision-making in CTSs is based on communication between human agents that often differ in technical competence (Knox et al., 2018). The point of communication is to establish a shared CSA between the analyst and the decision-maker such that the decision-maker can make good cyber defense decisions. This communication happens in the form of the analyst communicating a Recognized Cyber Picture (RCP) which is based on the analyst's CSA and contains carefully selected and actionable cyber threat information tailored to the needs of the recipient (Ahrend et al., 2016; Staheli et al., 2016; Ask et al., 2021a). A recent review of performance-related factors in SOC teams suggested that insufficient communication was among the biggest challenges faced by SOC team analysts but also one of the least researched topics (Agyepong et al., 2019). Another recent review (Ask et al., 2021a) that specifically looked at communication between humans in CTSs found that (a) there were no common best practices for information sharing; (b) technological aids (e.g., visualization tools and information sharing platforms) were not suited to fit the needs of the analysts; (c) there was a lack of studies simultaneously assessing individual- and team-level performance metrics; and (d) there was a general need for developing shared mental models for effective cyber threat communication.

In contrast to many other working environments, the personnel working within the cyber domain (NATO Cooperative Cyber Defense Center of Excellence, 2016) do not have direct sensory access to the space where events are taking place. In other words, when cyber personnel such as analysts are establishing CSA they are essentially trying to predict the future state of an environment they cannot directly observe. Instead, they are dependent on (1) tools that can detect and visualize events and activities in their cyber domain; and (2) their own mental models of that space. This may be a source of friction when relaying information between individuals because different individuals may have different mental models of the same phenomena, with corresponding differences in their understanding of the causal relationships contributing to those phenomena. This may affect what information different individuals think is important during a cyber threat situation (Ask et al., 2021a). For instance, previous research on the RCP needs of local- and national-level stakeholders in Sweden showed that no one listed knowledge about adversarial behavior as important for their RCPs (Varga et al., 2018). If awareness of adversarial behavior is required for achieving SA Level 1 during a CTS and is necessary to make good cyber defense decisions (Barford et al., 2009), then ignoring information of adversarial behavior may result in an insufficient CSA. Thus, stakeholders may have a mental model of causal relationships during a CTS that affect what kind of prioritizations they have and decisions that they make based on those prioritizations (Ask et al., 2021a).

While developing shared mental models have been suggested to ensure successful RCP communication during CTSs (Steinke et al., 2015; Ask et al., 2021a), little is known about the effect of visualization tools for cyber threat information communication and shared CSA such as how network topology is represented visually. The mammalian brain has evolved a neural architecture with an innate ability to process and understand information that relates to time and space (Eichenbaum, 2014; Ray and Brecht, 2016; Berggaard et al., 2018). Typical representations of network topology are in two dimensions (2D), which loses temporal and spatial relationships between nodes in the network, in addition to not scaling well with increased (but often necessary) complexity. Virtual Reality (VR) and Mixed Reality (MR) tools that are able to visualize CSA-relevant information such as network topology as 3D objects in space and time, may aid in the development of shared mental models for efficient RCP communication between technical and non-technical personnel (Kullman et al., 2018, 2019a,b, 2020). For instance, SA level 3 is the most vital stage for decision-making and appears to be the stage that is the most dependent on human working memory (Gutzwiller and Clegg, 2013). 3D visualizations of network topology in VR/MR may leverage automatic neurocognitive processes for encoding spatial information (Stackman et al., 2002; Angelaki and Cullen, 2008; Moser et al., 2008) when individuals are establishing a shared mental model of events in the network. If this allows CTS information to be encoded more efficiently (e.g., Legge et al., 2012; Wagner et al., 2021), it may also allow for more working memory capacity to be allocated to sharing knowledge about the course and impact of current and future events. Reducing the load on working memory may in turn support establishing shared SA level 3 (Gutzwiller and Clegg, 2013) for decision-making in CTSs (Kullman et al., 2019a).

Studies on VR navigation in humans and mice (Bohbot et al., 2017; Safaryan and Mehta, 2021) showed that they were able to generate brain waves in areas relevant for navigation, attention, learning, and memory (Winson, 1978; Seager et al., 2002). Similarly, previous VR research in humans showed that participants were able to use knowledge about the relationship between geometrical shapes in abstract space to navigate that space in a first-person VR navigation task (Kuhrt et al., 2021). This may further indicate that 3D visualizations that allow for exploring and interacting with network data in a way that facilitates spatial encoding of CSA information could leverage neurocognitive processes (Stackman et al., 2002; Angelaki and Cullen, 2008; Moser et al., 2008) that are currently underused in cyber defense.

**Figure 2:**
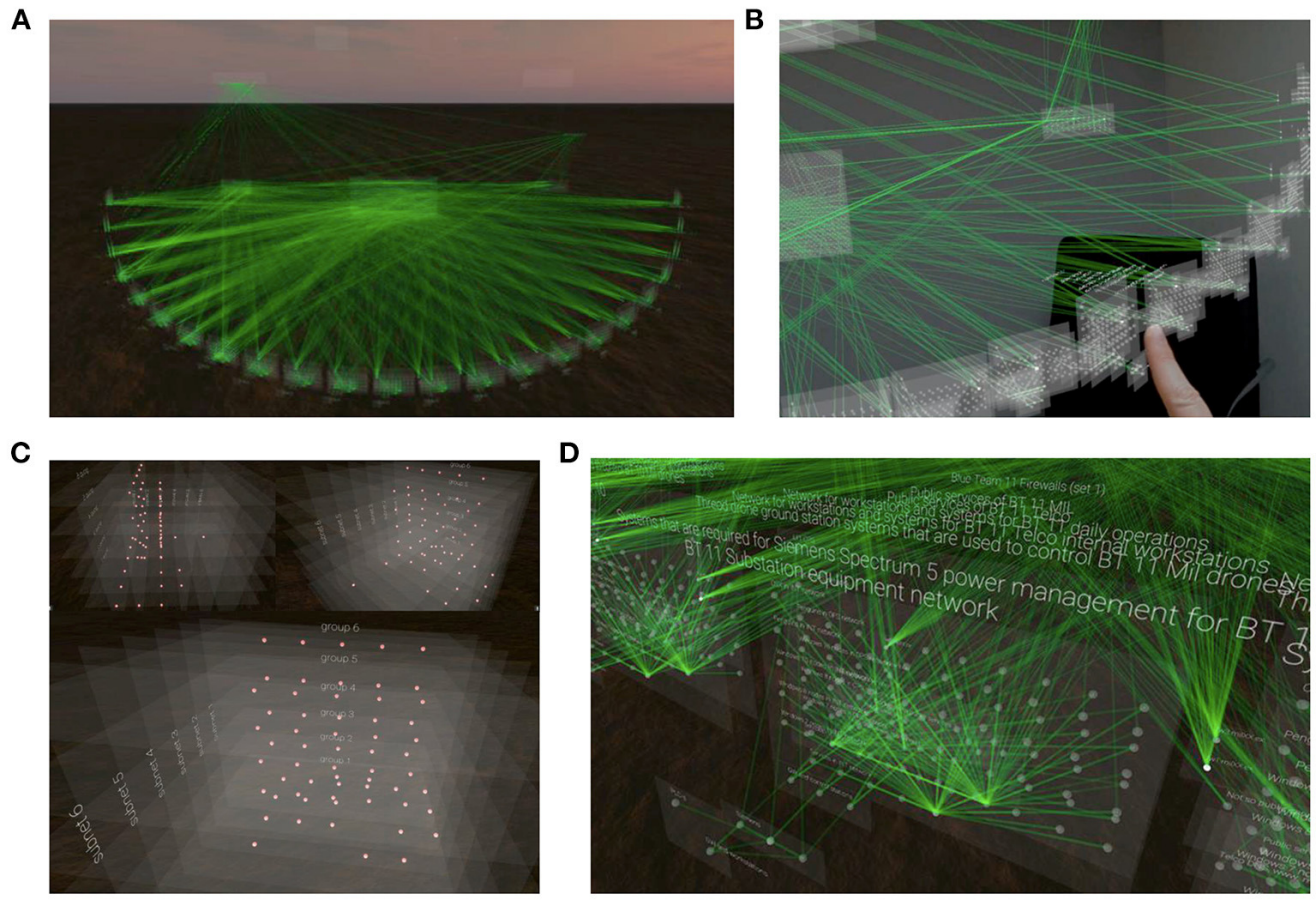
Visualization of network topography using the Virtual Data Explorer app. **(A)** Full overview of the metashape of the actual network that was used during the NATO CCDCOE 2018 Locked Shields event as visualized in VR using the VDE app. **(B)** An individual interacting with the network topography in MR. **(C)** A close-up of nodes in the network from different angles and without the edges representing the connections between them. **(D)** A close-up of Blue Team nodes in the network with descriptive information and the edges that connect them. Images taken from Kullman et al. (2019a).

The Virtual Data Explorer (VDE; Kullman et al., 2018, 2019a) was developed to visualize network topology in a manner that is idiosyncratic to the mental models that analysts use to conceptualize the network (Figure 2). Based on interviews with expert analysts, the VDE is able to visualize the relationship between nodes in an actual network in space and time (Kullman et al., 2018, 2019a,b, 2020). The visualizations produced by the VDE are interactive and can be shared between individuals, even remotely, thus allowing for collaborative development of shared mental models of events in the network. The VDE may therefore be a useful aid in the knowledge-transfer between technical and non-technical personnel such that shared CSA can be achieved to facilitate good cyber defense decision-making (Kullman et al., 2019a).

The VDE uses two distinct sets of information to visualize network topology: (1) the nodes included in a set of network traffic, and (2) mockup connections during a specified time-window or an attack path (Kullman et al., 2018). For the sake of clarity, we want to specify that the VDE is not a tool for carrying out forensic analyses. Instead, by visualizing network topology in time and space according to the mental model of the operator (Kullman et al., 2018, 2019a), the VDE may be a neuroergonomic tool for analysts to deepen their own understanding of how a CTS relates to the network they are tasked with defending, and for sharing CSA in complex working environments such as cybersecurity (Kullman and Engel, 2022a,b).

In the present study, we assess the effect of 3D visualization of network topology on communication and collaboration for CSA and cyber defense decision-making. The aim of this study is to determine if a 3D MR representation of a network attack, visualized by VDE is better than a 2D representation for (1) achieving Cyber Situational Awareness; (2) cyber team communication; and (3) decision-making among cooperating dyads during a simulated CTS.

## 2. Materials and methods

### 2.1. Ethics statement

This study was conducted under the Advancing Cyber Defense by Improved Communication of Recognized Cyber Threat Situations (ACDICOM) project.^1^ The present study conformed to institutional guidelines and was eligible for automatic approval by the Norwegian Social Science Data Services' (NSD) ethical guidelines for experimental studies. Participation was voluntary and all participants were informed about the aims of the study; the methods applied; that they could withdraw from participation at any time and without any consequences; and that, if they did so, all the data that was gathered from them would be deleted. After volunteering to participate in the study, participants were asked to provide informed consent on the first page of an online form where baseline data was collected. Participants were asked to generate and remember a unique participant ID that they would use during data collection for the duration of the study.

### 2.2. Participants and design

This experiment employed a pseudo-randomized head-to-head design using VDE in the experimental condition and the packet capture software Arkime (formerly Moloch) as the control condition. Participants (*N* = 22, mean age = 22.5, female = 5) were cyber cadets recruited from the Norwegian Defense University College, Cyber Academy (NDCA). Half of the cadets were specializing in military Information Communication Technology (ICT) systems while the other half were specializing in cyber operations.

The study consisted of two parts distributed over 3 days, where day one was used for gathering informed consent, and collecting demographic and baseline cognitive trait data. Results related to the cognitive data will be reported elsewhere. Day two and three was used for the experiment. After providing informed consent and filling out initial questionnaires, participants were randomized in dyads and allocated to either the VDE or the Arkime condition. During the experiment, dyads had to collaborate to familiarize themselves with the network topology and to identify indicators of compromise. The participants in the VDE condition used HoloLens 2 (Microsoft) for the MR visualizations of network topology as their only aid. The participants in the Arkime condition also had a 2D schematic illustration of the network topology available to them in paper format.

The network topology and activity used for this experiment was visualized using network data from the 2022 Locked Shields Cyber Defense Exercise (CDX) provided by the NATO Cooperative Cyber Defense Center of Excellence (CCDCOE). The experiment lasted for approximately 2 h per dyad.

### 2.3. HoloLens 2

Microsoft HoloLens 2 (Microsoft, Redmond, DC) has become the most common MR headset to be used for various research studies, fielded by enterprises and governments for Interactive Stereoscopically Perceivable Multidimensional Data Visualizations (ISPMDV; see Kullman and Engel, 2022b for an introduction), where its mostly used for either geospatial or natively spatial datasets. For the purposes of this study, HoloLens 2 was chosen for its capabilities, ease of software development, and existing compatibility with VDE.

### 2.4. The Virtual Data Explorer and visualization of network topology

VDE (Kullman et al., 2018, 2019a,b, 2020; [https://coda.ee/]) enables a user to perceive the spatial layout of a dataset, for example the topology of a computer network, while the resulting ISPMDV (Kullman and Engel, 2022a,b) can be augmented with additional data, like TCP/UDP session counts between network nodes. Users can customize ISPMDV layouts using textual configuration files that are parsed by a VDE Server and used while showing the visualization by a VDE Client.

VDE functionality is decoupled to server and client components in order to accommodate timely processing of large query results (from the user's dataset) in a more powerful environment (than a wireless MR headset) before data is visualized either by a VR or MR headset. The VDE Server also acts as a relay to synchronize the visualizations (e.g., grabbed objects position in connected users' views) between connected users' sessions so that a connected user's actions manipulating a visual representation of data can be synchronized with other connected users working with that same dataset.

Only a subset of VDE capabilities was employed in the present study: the dataset was preloaded to the headset along with the application (to avoid any possible networking related issues) while VDE Server was used only to facilitate multi-user sessions.

A previous study indicated that there was a need for more experimental collaboration between cognitive scientists and CDX organizers (Ask et al., 2021a). For this study, a NATO CCDCOE Locked Shields 2022 CDX Blue Team's network topology was visualized for the participants with VDE and overlaid with edges (network session counts) between cubes (networked entities). Within view at any given time (depending on user's location and direction) were up to 958 nodes and groups, with up to 789 edges.

**Figure 3:**
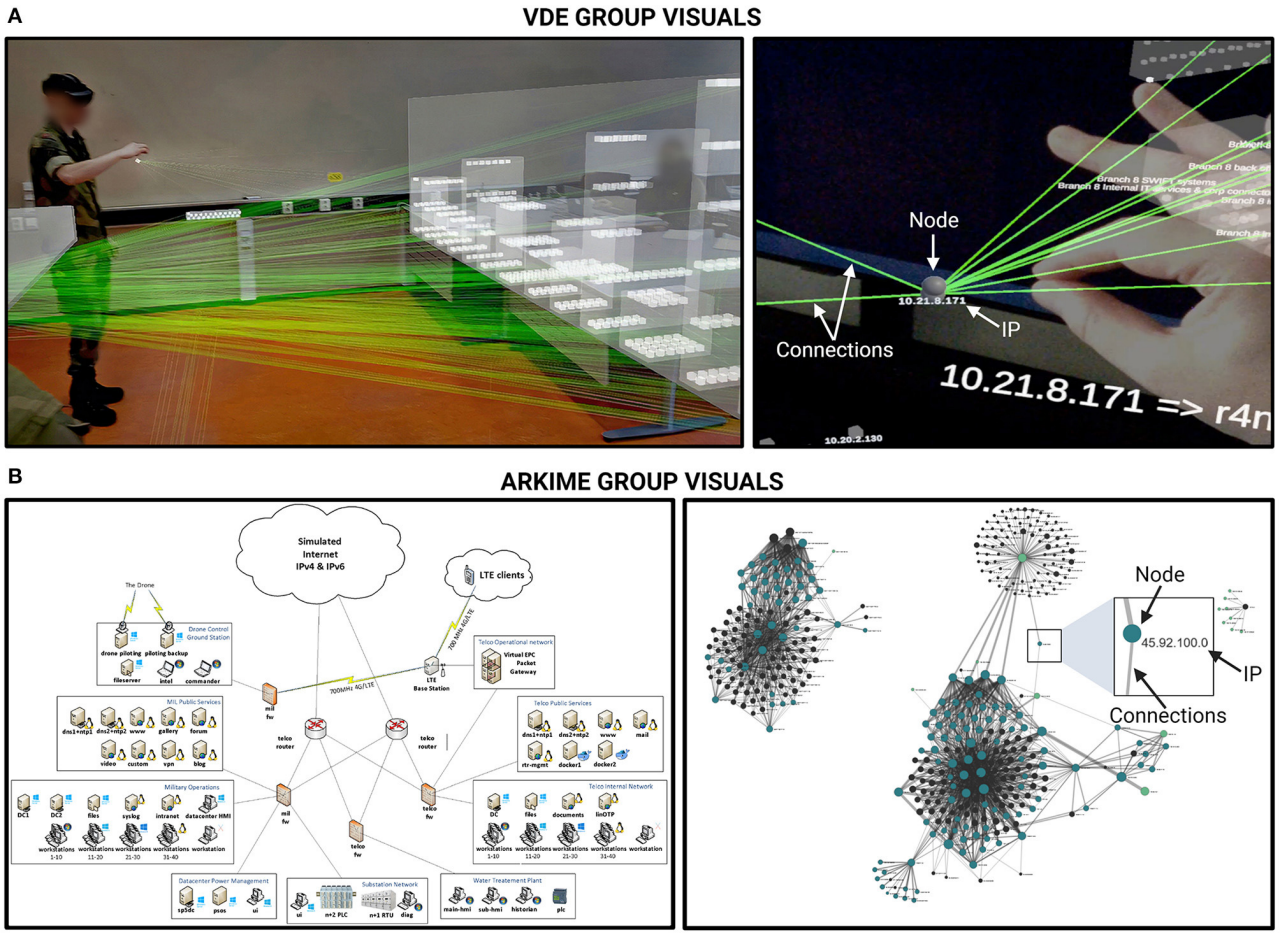
Overview of the visualization tools used in each condition. **(A)** The Virtual Data Explorer (VDE) representation of the network topology. The first image in the panel (left-hand side) depicts an overview of the network layout used in the present study. The second image (right-hand side) is a representative close-up (taken from Kullman and Engel, 2022a). White arrows have been superimposed on the image on the right to indicate node/hosts, edges that represent connections between nodes, and the host IP address. **(B)** Images depicting the 2D network topology as shown in the Arkime condition. The first image in the panel (left-hand side) depicts an approximation of the 2D representation of network topology as shown in the paper schematics. The second image (right-hand side) depicts a graph representation of the network topology as shown in the Arkime software, where dots are hosts and edges are the connections between them. Participants could zoom in, select nodes to see exclusive connections, session number, and so on. Black arrows have been superimposed on the image on the right to indicate node/host, edges that represent connections between nodes, and the host IP address.

All study participants perceived the ISPMDV being positioned in the same location and direction in the room where the study was conducted (see Figure 3A, image on the left). Participants did not have the capability to reposition the visualization components permanently, but they could grab (pinch) a node to better understand its connections while temporarily moving the node around. Once the participant let go of the node, it returned smoothly to its initial location.

As the study participants did not have prior knowledge of Locked Shields 2022 networks and topology, the topology visualization they experienced was not created based on their mental models (as would be the suggested course of using VDE after employing mental model mapping method for cybersecurity; Kullman et al., 2020). Instead, the participants received an introduction about the topology as described in the task one procedures (Section 2.7.1.).

### 2.5. Arkime packet capture software

Arkime (v3.4.2 [https://arkime.com/]) was used for preparing the dataset from Locked Shields 2022 network traffic both for the VDE ISPMDV view, as well as for the comparative group that used 2D and textual information. Participants were given access to an Arkime instance and taught the basics of using its interface (Sessions and Connections tabs). In the Connections tab, participants had a 2D graph view (see Figure 3B, image on the right) onto the exact same set of nodes and edges that VDE participants had with HoloLens. When participants hoovered over the edges connecting nodes (hosts) to each other, the amount of traffic was displayed on a left-hand panel as described in the task two procedures (Section 2.7.2.).

### 2.6. Hardware functionality and operational stability

The HoloLens 2 headsets had a tendency to overheat after a period of use, upon which the Windows Operating System running the headset froze the VDE application. This left the network visualization flickering in the user's view. As this issue only started to manifest during the second half or the 1st day of the study, we suspected that the problem originated from thermal issues. To keep the study going, we relied on three HoloLens 2 headsets, of which two were used by a dyad on the floor while the third one was being charged. Rapid charging and then discharging while the headset's GPU and CPU were being heavily utilized by the VDE application seemed to have been too much for the headset's thermal dissipator. Switching a participant's malfunctioning headset during a trial was sub-optimal, hence we needed a more sustainable setup. The solution for the HoloLens 2 overheating problem was to use power delivery capable battery packs. The setup on the 2nd day was for the users to wear the headsets, while having battery packs in their pockets that were connected to the headsets with power delivery capable cables. This allowed the headsets to be used uninterrupted for the duration of a given dyad's trial.

### 2.7. Procedure

**Figure 4:**
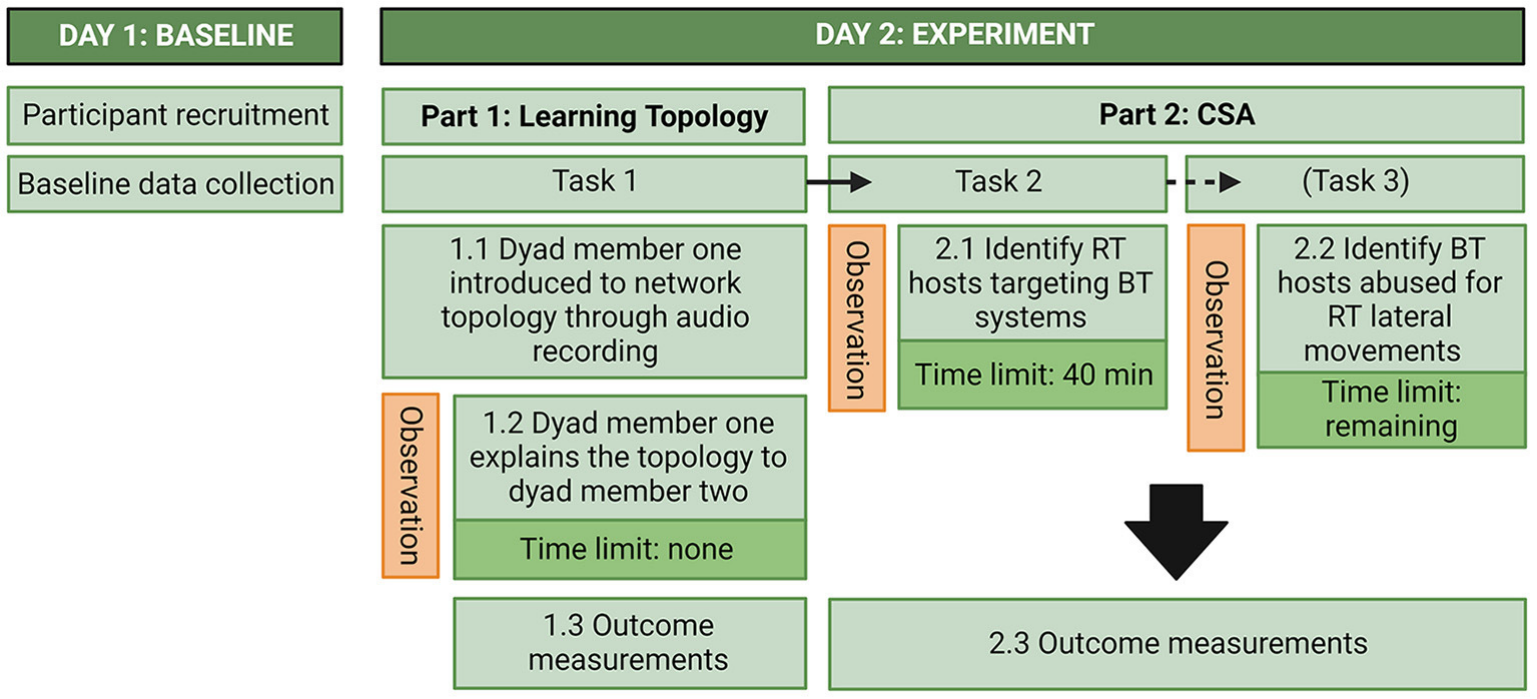
Overview of the experiment. RT, Red Team; BT, Blue Team; CSA, Cyber Situational Awareness; VDE, Virtual Data Explorer.

The study was conducted at the NDCA. The two experimental conditions were conducted in parallel, one dyad at a time, and in separate rooms that were secluded from other activities. The experiment consisted of two parts. In the first part, one participant from each dyad was introduced to the network topology which they then had to explain to the other participant in the dyad. In the second part, participants in each dyad had to collaborate to identify indicators of compromise. Measurements were done thrice; baseline measures upon arrival and then outcome measures after each part of the experiment. For the outcome measures after each part, participants filled out questionnaires assessing task success, confidence in answers, and how they experienced communicational, coordination, emotional, and performance monitoring load related to their teamwork. After part two the participants also had to answer some CSA-related questions that they were not explicitly asked to solve in the task instructions they were given. During the experiment, verbal communication and the time dyads spent on finishing each task was scored by observers. Figure 4 shows an overview of the study and each part of the experiment.

#### 2.7.1. Task one: Understanding the network topology

Upon arriving at the experiment, both participants in the dyad were given a link to the online form which they accessed with laptops. The dyads in the VDE condition spent a few minutes having the HoloLenses they were going to use calibrated to their eyes before filling out the questionnaires. The dyads were referred to as teammates for the duration of the experiment.

After filling out the questionnaires related to the baseline-measurements the form presented a prompt telling the participant to pause and wait for instructions. After both participants were done filling out the questionnaires, one participant was asked to wait outside the room until summoned by the experimenter. The other participant in the dyad was then either told to put on the HoloLenses (if in the VDE condition) to see the 3D representation of the network topology or seated at a table where the 2D schematics of the network topology was depicted (Arkime condition).

Upon confirming that they saw a network in front of them, the participants were played an English audio recording explaining that what they saw was the network that the Blue Team had to defend during the Locked Shields 2022 CDX. The recording lasted for 3 min and 30 seconds. It was explained to them what nodes each segment in the network consisted of, what was considered normal activity, where known Red Team nodes were, and which nodes were unknown. In the VDE condition, the participant was instructed to walk through the nodes and also how to interact with the nodes to probe for further information (e.g., touch node to see the IP address or pinch node to lift up in order to see which nodes it was connected to).

The briefing was only given once (which was stated in the beginning of the recording). After the recording was over, the participant was given the instruction that their task would be to explain the network topology to their teammate. They were instructed to get confirmation from their teammate that they had understood the topology upon which they would either (1) re-explain if their teammate did not understand or (2) let the experimenter know that they had completed the task. After confirming that they had understood the task, the other participant in the dyad was summoned and then the first participant was told to start at their convenience. In the VDE condition, the participant that was summoned was told to put on their HoloLens and confirm that they saw the network representation in front of them before the first participant in the dyad was given the signal to start. There were no time constraints on this task.

After signaling that the task was over, the participants were instructed to access their laptops and continue filling out the questionnaires until getting to a prompt asking them to wait for further instructions. In the VDE condition, the participants were instructed to remove their HoloLenses while answering the questionnaires.

#### 2.7.2. Task two: Identifying Red Team hosts targeting Blue Team systems

After both participants were done filling out the questionnaires, they received the instructions for the second task. In the VDE condition, both participants were told to put on their HoloLenses again. This time the 3D representation of the network topology was updated with more edges connecting each node. The edges varied in brightness depending on the number of sessions (traffic) associated with the connections.

In the Arkime condition, both participants were introduced to a graph representation of the Blue Team network topology in Arkime. They were instructed (1) that they could select nodes to see their associated IP addresses and communications targets (represented by edges between the nodes); and (2) that they could see the session count (amount of traffic) associated with each connection by hovering over the edges connecting each node. The edges varied in thickness depending on the amount of traffic associated with the connection.

The dyads were then instructed to collaborate to find the top five Red Team hosts (nodes) targeting Blue Team systems according to the amount of traffic associated with each connection. For this task, they were given pen and paper to note the IP address associated with each identified Red Team host. The dyads were instructed to confirm with each other when they were done with the task before notifying the experimenter.

Both conditions saw the same network, with the same number of nodes and edges and the same amount of traffic. Participants in the VDE condition could not see the session count associated with each connection but could only use the edge brightness as cue. The participants had 40 min to finish the task, although this was not disclosed to them.

Upon notifying the experimenter that they had finished the task, the participants were given the instructions for the third task. If the time ran out before a dyad could finish the task, they were stopped by the experimenter and told to finish the last set of questionnaires.

#### 2.7.3. Task three: Identifying Blue Team hosts abused for Red Team lateral movements

For the third task, the dyads were instructed to collaborate to find evidence, if any, of Red Team lateral movements and to note down the top five Blue Team hosts that were possibly abused for that purpose according to the amount of traffic associated with the connection.

The dyads were told that they had a time limit and what the duration of that time limit was (which was the time remaining from the 40 min they had to finish the previous task). As for the previous task, they were instructed to confirm amongst each other that they had finished the task before signaling to the experimenter that they were done.

After completing the task (or if the time ran out), the dyads were instructed to complete the last set of questionnaires. This was done individually. They were allowed to look at their notes from tasks two and three when answering questions about hosts and IP addresses but were not allowed to communicate or collaborate when answering the questionnaires.

After the Arkime group was done with the experiment, they also did the first task of the VDE condition, receiving instructions as described previously. The roles for task one were the same as in the Arkime condition, meaning that the participant who explained the topology to their teammate in the Arkime condition also did so in the VDE condition. Initially, we wanted the Arkime group to run through the entire experiment in the VDE condition as well. Due to time constraints and the experiment needing to be conducted on the same day, this was limited to completing the first task. Data related to these measurements will be reported elsewhere.

### 2.8. Data measures

#### 2.8.1. Understanding the network topology

Per definition (Endsley, 1988; Franke and Brynielsson, 2014), to acquire CSA during a CTS in a cyber environment, one must necessarily know the normal state of the environment. To assess the participants' understanding of the network topology, we used a questionnaire partly inspired by the CSA for Analysts Questionnaire (Lif et al., 2017). The CSA questionnaire asks participants to draw a description of the network topology with sources and targets of attack. As our measurements were collected digitally, we employed a forced choice questionnaire where participants had to choose the one of four images that had the most correct 2D depiction of the network topology they had reviewed. The images varied in how connections between Blue Team segments were depicted, while some network segments were missing from the incorrect topology images. To avoid problems with resolution, the images were numbered and presented on laminated A3 paper while the participant provided their answers in the online form. Correct answers were scored as 1 and incorrect answers were scored as 0.

Our initial plan was to have two sets of forced choice questionnaires (in two different formats) that both conditions had to answer. One set would include the 2D schematics that were used in the forced choice questionnaire administered in the present study, while the other set of network topology images would be based on the 3D representation in VDE. Each set of questionnaires would therefore favor the condition where the format matched the condition (e.g., the 2D images favor the Arkime condition where 2D representations of the network topology is part of the tools available to the participants). The idea was that, if one condition performed better on the forced choice set that favored the other condition, this would say something about the level of understanding of the network topology that the participants were able to extract from either the 2D schematics or the VDE representation. However, due to time restraints, we could only use one set of forced choice questionnaires. As the current forced choice questionnaire favors the Arkime condition, it also serves as a test for whether the VDE representation induces overconfidence if the VDE group performs worse on this test than the Arkime group but is more or equally confident in their answers.

#### 2.8.2. CSA item 1: Adversarial behavior

To assess the outcome of task two, one of the items asked: “What are the possible Red Team hosts that were targeting the Blue Team systems?”. The participants had to write down the five IP addresses that they identified during task two. The answers were used to generate three variables: (1) total number of hosts identified, (2) total number of correctly identified hosts, and (3) total number of sessions associated with correctly identified hosts.

#### 2.8.3. CSA item 2: Impact of the attack

To further assess the participants' CSA, they were asked to “Choose Blue Team segments in which the Red Team has been trying to compromise Blue Team hosts”. For this item, the participants were given a multiple-choice questionnaire listing five Blue Team segments that were possibly affected. The participants could choose as many as they wanted. Because all of the segments were affected, answers on this item were scored by adding up all the segments that were chosen by the participants giving a numerical score ranging from 0 (the minimum of correct answers) to 5 (the maximum of correct answers).

#### 2.8.4. CSA item 3: Situational report

To assess their comprehension of the cyber threat situation (awareness of the current situation, what caused it, and how it may evolve), participants were asked to answer three qualitative, open-ended questions. The questions were taken from a SITREP developed by one of the co-authors for use in cyber defense exercises. The questions included: “(1) Describe the activity you saw (specific but not overly detailed)”, “(2) What type of incident do you think it was?”, and “(3) If you could suggest anything - which actions should be done?”.

The answers were blinded and scored individually by one of the co-authors who participated at Locked Shields 2022 exercise and had access to the ground truth of the dataset used. The answers were scored on a 5-point scale ranging from 0 (not correct/irrelevant) to 1 (correct/relevant). The answers were given an overall k-score ranging from 0 (not thorough) to 9 (thorough) to indicate the level of thoroughness combined in the answers to all three questions.

#### 2.8.5. CSA item 4: Adversarial behavior and impact of attack

To measure the outcome of task three, participants were asked: “If any, what were the indicators of Red Team lateral movements in Blue Team networks? Name BT hosts that were (possibly) (ab)used for that purpose.” The participants had to write down the IP addresses that they identified during task three. Answers on this item were used to generate three variables: (1) total number of hosts identified, (2) total number of correctly identified hosts, and (3) total number of sessions associated with correctly identified hosts.

Because the information required to solve task three was available to all participants at all times from the initiation of task two, all participants had to answer this item regardless of whether they were given the opportunity to solve task three or not.

#### 2.8.6. Confidence in answers

After each question, participants were asked to rate how confident they were in their answers on a 11-point scale ranging from 0 to 100%.

#### 2.8.7. Decision-making forced-choice task

To assess the effect of condition on decision-making, participants were asked to answer a forced-choice decision-making question with four possible alternatives. The item asked: “If you could only pick one course of action, which would you pick?”. The four alternatives were: (1) Cut off all connectivity from the friendly networks to outside, (2) Start incident response on selected hosts, (3) Launch attacks against the hosts that the suspected adversaries might be using, or (4) Cut off connectivity to a selection of network segments. An additional question was asked: “If you picked 4, what would be your suggested network segments?”. Each choice was used to generate four variables scored as 0 (not chosen) and 1 (chosen).

#### 2.8.8. Team workload questionnaire (select items)

The Team Workload Questionnaire (TWLQ; Sellers et al., 2014) was used to assess how participants experienced workload demands on team tasks during the exercise. The items are scored on an 11-point Likert scale ranging from very low to very high. High scores indicate higher levels of subjective workload. The TWLQ consists of six subscales divided on two dimensions, the Teamwork component (communication, coordination, team performance monitoring) and Task-Team component (time-share, team emotion, team support). For the purpose of the present study, we were mainly interested in the communication demand item as an indicator of whether the VDE would reduce communication demands. We were also interested in the items related to coordination demand, demand for controlling their own emotions, and demand for monitoring their own performance. The four TWLQ items were administered two times; the first at the end of task one and the second at the end of the experiment.

#### 2.8.9. Structured observation

Structured observation was performed to assess the frequency of occurrence for four verbal communication behaviors: (1) Orient, Locate, Bridge (OLB) processes, (2) perceptual shared mental modeling, (3) task resolution, and (4) communication dysfunction.

OLB behaviors included communication behaviors related to perspective taking and grounded communication to achieve a shared understanding of the situation in accordance with the OLB model (Knox et al., 2018). Some examples included when members of the dyads asked questions to probe each other's understanding of what was communicated; adjusted language (from technical to less technical) to make sure the recipient understood the significance of what was communicated; and gave each other updates to maintain a mutually shared overview of what they were doing and discovering at any given moment.

Perceptual shared mental model behaviors included verbal communication related to achieving a shared perception of anything related to the task. Examples included utterances such as “Come here and look at this,” “When I stand here I see x,” “Do you see this node? It is communicating with that node over there,” and so on.

A previous observational study indicated that team communication related to task resolution was different between well- and poor-performing teams during a CDX (Jariwala et al., 2012). In our study, task resolution behaviors included verbal communication related to the status or completion of the specific tasks that they were assigned. Examples included participants in the dyad asking each other “How many hosts have we found now?”, “How many hosts did we have to find again?”, and “Should we say that we have completed the task?”.

Communication dysfunction behaviors included communication where participants in the dyad talked over/interrupted each other, did not answer each other's questions, argued, went too long (over 2 min) without communicating, and so on. Examples included instances where a participant started explaining what they were seeing and the other participant interrupting them to talk about what they were seeing.

Two observers/coders, one per condition, were used for the scoring of items. Score per dyad was determined by noting frequency of behavioral occurrence during the experiment. The coders agreed how to categorize the behaviors prior to the experiment, and the same coders were used throughout the experiment to ensure reliability. To assess inter-rater reliability, both observers simultaneously scored one of the dyads followed by performing a two-way mixed, absolute, single measures intra-class correlation (ICC) analysis on the raw scores for each item (Shrout and Fleiss, 1979; Hallgren, 2012). Inter-rater reliability was excellent (ICC = 0.871; Cicchetti, 1994). The observers also noted the time (minutes) spent to finish each task.

#### 2.8.10. User experience measurements

To measure the experience participants had with using the HoloLens 2 and the VDE, we administered the User experience in Immersive Virtual Environment questionnaire (Tcha-Tokey et al., 2016). This data will be reported elsewhere.

#### 2.8.11. Cognitive tests and self-report measures

We collected a range of cognitive trait and state data including measurements that have been identified as relevant for performance in previous studies on cyber cadets and cyber security personnel (Knox et al., 2017; Lugo and Sütterlin, 2018; Jøsok et al., 2019; Ask et al., 2021b; Sütterlin et al., 2022). For instance, positive moods and overconfidence has been found to be associated with poorer metacognitive judgments of CSA during a cyber engineering exercise (Ask et al., 2023), and in detecting cyber threats not directly related to network intrusion (Sütterlin et al., 2022). Conversely, self-regulation abilities measured through self-report and neurophysiological indicators were found to predict cognitive flexibility in terms of mental context shifting during a cyber defense exercise (Knox et al., 2017; Jøsok et al., 2019) and better metacognitive judgements of performance (Ask et al., 2023), respectively. Furthermore, metacognition, self-regulation, and cognitive flexibility are necessary for establishing and communicating CSA (Jøsok et al., 2016; Knox et al., 2018; Endsley, 2020; Ask et al., 2023). Cognitive data was collected with tests and self-report questionnaires on both days of the experiment. The cognitive data collected on day one included cognitive styles, cognitive flexibility, emotion regulation, vividness of mental imagery, and rumination. The cognitive data collected during the experiment included affective states (baseline) and metacognition (projections for how well they thought they would perform at baseline and correction of how well they thought they had performed after the experiment was over). As noted, the results related to the cognitive data will be reported elsewhere.

### 2.9. Data analysis

The data were summarized and presented in tables using mean (M) and standard deviations (SD) for continuous and numerical variables, and frequency (count) and percentage (%) for ordinal variables.

The Shapiro-Wilk test of normality and confirmatory visual inspection revealed that most variables were not normally distributed. The exceptions included part one communication demands, part one coordination demands, part one performance monitoring demands, confidence in CSA 1 answers, confidence in CSA 3 descriptions, part two emotion demands, part two performance monitoring demands, and task two OLB. Non-parametric tests were performed for all subsequent analyses except for those variables.

For the non-parametric analyses, the Kruskal-Wallis *H* test was used for comparisons between the VDE group and the Arkime group. Results were presented as *H* statistic (degrees of freedom; df), *p*-values, and effect size. Effect size (η^2^) for Kruskal-Wallis *H* test was calculated as (*H* – *k* + df)/(*n* – *k*); where *H* was the Kruskal-Wallis statistic, k was the number of groups, and n was the total number of observations (*n* = 22). Dunn's *Post-Hoc* test was used to assess significant relationships for non-parametric variables between groups and was reported as z-statistic and Bonferroni adjusted *p*-values (*p*_bonf_).

For the parametric analyses, one-way ANOVAs were performed. Results for ANOVA were reported as *F* statistic(df), *p*-values, and effect size. Effect size (ω^2^) for ANOVA was calculated as [sum of squares between − (*k* – 1) mean square within]/(sum of squares total + mean square within). Tukey's *post hoc* test was used to assess significant relationships for parametric variables between groups and was reported as mean difference (MD) and *p*_bonf_.

Between-group differences were visualized in interval plots with 95% confidence intervals.

The relationship between communication variables that were significantly different between the groups and CSA variables that were significantly different between the groups were assessed with Spearman correlation (2-tailed) on z-transformed variables. Results were visualized in a heat map and presented as correlation coefficients (ρ) and *p*-values. Separate regression analyses were performed for significant relationships. Results were reported as standardized beta (β), *p*-values, adjusted R^2^ ($R_{\text{Adj}}^{2}$), and *F*(df) statistics.

Alpha levels for hypothesis testing were set at the 0.05 level for all analyses. All data were analyzed using JASP version 0.15 (JASP Team, 2021).

## 3. Results

Table 1 presents descriptive statistics of participant characteristics and experimental outcome measurements.

**Table 1:** Descriptive statistics (*N* = 22).

	**Total**			**VDE**			**Arkime**		
**Variables**	**M**	**SD**	**Count (%)**	**M**	**SD**	**Count (%)**	**M**	**SD**	**Count (%)**
Age	22.59	1.36		22.50	1.44		22.70	1.33	
Gender (male)			17 (77.27)			7 (58.33)			10 (100.00)
Military IT systems			13 (59.01)			8 (66.66)			5 (50.00)
Cyber operations			9 (40.90)			4 (33.33)			5 (50.00)
**Part 1**									
Select correct image	0.59	0.50	13 (59.09)	0.33	0.49	4 (33.33)	0.90	0.31	9 (90.00)
Confidence in choice	61.36	35.49		41.66	33.25		85.00	21.21	
Communication demand	5.90	1.95		5.91	1.73		5.90	2.28	
Coordination demand	4.90	1.82		5.25	1.60		4.50	2.06	
Emotional demand	3.81	3.01		3.66	2.93		4.00	3.26	
Performance monitoring demand	5.13	2.03		4.66	1.96		5.70	2.05	
**Part 2**									
CSA 1 total RT hosts	3.36	1.62		4.41	1.16		2.10	1.10	
CSA 1 correct RT hosts	2.59	2.01		4.00	1.34		0.90	1.19	
CSA 1 RT hosts total traffic	26525.77	28681.35		48583.25	20069.14		56.80	104.19	
CSA 1 confidence	41.81	26.30		54.16	23.14		27.00	22.63	
Finished task 2 < 40 min	0.72	0.56	16 (72.72)	0.66	0.49	8 (66.66)	0.80	0.42	8 (80.00)
CSA 2 total BT segments	1.59	0.73		2.00	0.73		1.10	0.31	
CSA 2 confidence	40.90	29.09		52.50	26.32		27.00	27.10	
CSA 3 SITREP—activity	0.62	0.36		0.77	0.31		0.45	0.35	
CSA 3 SITREP—incident	0.60	0.42		0.72	0.40		0.45	0.40	
CSA 3 SITREP—actions	0.52	0.42		0.60	0.44		0.42	0.39	
CSA 3 SITREP—K-score	5.04	3.25		6.16	3.29		3.70	2.79	
CSA 3 confidence	38.63	22.52		47.50	19.59		28.00	22.01	
CSA 4 total BT hosts	0.96	1.61		1.50	1.97		0.30	0.67	
CSA 4 correct BT hosts	0.81	1.53		1.33	1.87		0.20	0.63	
CSA 4 BT hosts total traffic	640.54	1086.05		732.50	1040.58		530.20	1184.88	
CSA 4 confidence	40.90	32.05		56.66	27.08		22.00	27.80	
Communication demand	7.63	0.84		7.33	0.77		8.00	0.81	
Coordination demand	6.72	1.77		6.50	2.23		7.00	1.05	
Emotional demand	4.63	2.59		4.41	2.93		4.90	2.23	
Performance Monitoring demand	6.09	2.11		5.91	2.39		6.30	1.82	
**Forced decision-making**									
Decision 1	0.04	0.21	1 (4.54)	0.08	0.28	1 (8.33)	0.00	0.00	0 (0.00)
Decision 2	0.90	0.29	20 (90.90)	0.83	0.38	10 (83.33)	1.00	0.00	10 (100.00)
Decision 3	0.00	0.00	0 (0.00)	0.00	0.00	0 (0.00)	0.00	0.00	0 (0.00)
Decision 4	0.04	0.21	1 (4.54)	0.08	0.28	1 (8.33)	0.00	0.00	0 (0.00)

CSA, Cyber situational awareness; RT, Red team; BT, Blue team; SITREP, Situational report.

### 3.1. The effect of VDE on cyber situational awareness

#### 3.1.1. Baseline network topology recognition

Kruskal-Wallis *H* test was performed to assess the differences of condition on task one outcome variables. Table 2 shows the results of the comparisons between the VDE group and the Arkime group on selecting the correct image depiction of the network topology, confidence in image selection, and TWLQ item responses.

**Table 2:** Task 1 comparisons between VDE and Arkime (*N* = 22).

	**Kruskal-Wallis test**			**Dunn's** ***post hoc***	
**Variables**	***H*** **(1)**	***p***	η^2^	**z**	*p*_bonf_
Select the correct image	6.916	**0.009**	0.295	−2.630	**0.004**
How confident are you about this?	7.469	**0.006**	0.323	−2.733	**0.003**
Emotional demand	0.059	0.808	−0.047	-	-
	**One-way ANOVA**			**Tukey's** ***post hoc***	
	***F*** **(1)**	***p***	ω^2^	**MD**	*p*_bonf_
Performance monitoring demand	1.442	0.244	0.020	-	-
Communication demand	0.000	0.985	0.000	-	-
Coordination demand	0.919	0.349	0.000	-	-

η^2^, Effect size; *p*_bonf_, Bonferroni adjusted *p*-values; Bold, significant differences; ω^2^, Effect size; MD, Mean difference.

The Kruskal-Wallis test showed that the VDE group selected the correct network topology image significantly different from the Arkime group (*p* = 0.009). Dunn's *post hoc* test showed that the VDE group selected the correct network topology image significantly less than the Arkime group (z = −2.63, *p*_bonf_ = 0.004).

The Kruskal-Wallis test showed that the confidence in image selection was significantly different between the VDE group and the Arkime group (*p* = 0.006). Dunn's *post hoc* test showed that the VDE group was significantly less confident in their image selection than the Arkime group (z = −2.73, *p*_bonf_ = 0.003).

No significant differences were observed on any of the TWLQ items measured after the completion of task one.

#### 3.1.2. Red team movements, attack severity, and situational reports

Kruskal-Wallis *H* test was performed to assess the differences in the effect of condition on task two and three outcome variables. Table 3 shows the results of the comparisons between the VDE group and the Arkime group on identifying Red Team hosts targeting Blue Team systems, identifying affected blue team segments, assessment of the observed activity, assessment of what incident it was, suggestions of what actions to do as response, identifying Blue Team hosts abused for Red Team lateral movements, confidence in responses, and TWLQ item responses.

**Table 3:** Comparison of task two and task three results between VDE and Arkime (*N* = 22).

	**Kruskal-Wallis test**			**Dunn's** ***post hoc***	
**Variables**	***H*** **(1)**	***p***	η^2^	**z**	*p*_bonf_
CSA 1. Number of identified possible RT hosts that were targeting the BT systems	11.603	**<0.001**	0.530	3.406	**<0.001**
CSA 1. Correctly identified RT hosts that were targeting the BT systems	12.867	**<0.001**	0.593	3.587	**<0.001**
CSA 1. Correctly identified RT hosts that were targeting the BT systems—traffic total	15.822	**<0.001**	0.741	3.978	**<0.001**
CSA 2. Compromised BT Segments correctly identified	8.863	**0.003**	0.393	2.977	**0.001**
CSA 2. How confident are you about this?	4.121	**0.042**	0.156	2.030	**0.021**
Finished task 2 on time	0.467	0.495	−0.026	-	-
CSA 3. SITREP—Describe the activity you saw	4.035	**0.045**	0.151	2.009	**0.022**
CSA 3. SITREP—What incident do you think it was?	2.743	0.098	0.087	-	-
CSA 3. SITREP—Which actions should be done?	0.988	0.320	−0.000	-	-
CSA 3. SITREP—Thoroughness K-score	3.044	0.081	0.102	-	-
CSA 4. Total BT hosts abused for RT lateral movements	1.735	0.188	0.037	-	-
CSA 4. Correctly identified BT hosts abused for RT lateral movements	3.681	0.055	0.134	-	-
CSA 4. BT hosts abused for RT lateral movements—Traffic	0.515	0.473	−0.024	-	-
CSA 4. How confident are you about this?	6.651	**0.010**	0.282	2.579	**0.005**
Communication demand	3.919	**0.048**	0.145	−1.980	**0.024**
Coordination demand	0.029	0.866	−0.048	-	-
	**One-way ANOVA**			**Tukey's** ***post hoc***	
	***F*** **(1)**	***p***	ω^2^	**MD**	*p*_bonf_
CSA 1. How confident are you about this?	7.667	**0.012**	0.233	27.458	**0.012**
CSA 3. SITREP—How confident are you about the descriptions above?	4.832	**0.040**	0.148	19.500	**0.040**
Emotion demand	0.182	0.674	0.000	-	-
Performance monitoring demand	0.172	0.682	0.000	-	-

η^2^, Effect size; *p*_bonf_, Bonferroni adjusted *p*-values; Bold, significant differences; CSA, Cyber situational awareness; RT, Red Team; BT, Blue Team; SITREP, Situational report; ω^2^, Effect size; MD, Mean difference.

Two dyads, one from VDE group and one from Arkime group, spent >40 min on exploring the topology in task one. The dyad in the VDE group spent the least amount of time of all dyads on finishing task two (15 min). The dyad in the Arkime group could not finish task two in < 40 min. The maximum amount of time spent to finish task two was 35 min. Thus, the amount of time the dyads had to finish task three ranged from five to 25 min.

There were no significant differences between the groups with respect to finishing task two within the 40-min time limit (*p* = 0.495). In general, the VDE group had higher scores than the Arkime group on all performance outcomes and lower scores on all team workload measures during the second part of the experiment, although not all of these differences were significantly different between the groups.

#### 3.1.3. CSA 1: Identifying RT hosts targeting BT systems

The Kruskal-Wallis *H* test showed that the total number of identified Red Team hosts targeting Blue Team systems was significantly different between the VDE and the Arkime group (*p* < 0.001). Dunn's *post hoc* test showed that the VDE group identified significantly more Red Team hosts targeting Blue Team systems compared to the Arkime group (z = 3.40, *p*_bonf_ < 0.001).

**Figure 5:**
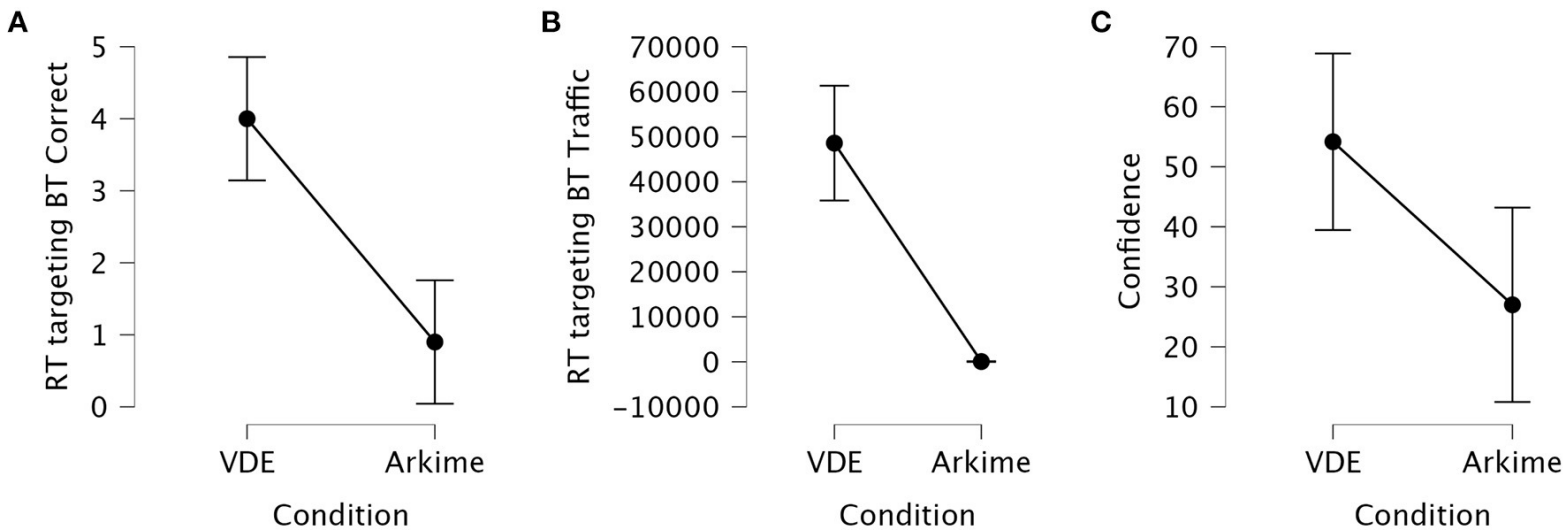
Interval plots for the differences in identifying Red Team hosts targeting Blue Team systems. **(A)** Correctly identified Red Team hosts. **(B)** Traffic associated with correctly identified Red Team hosts. The number of sessions associated with correctly identified hosts ranged from 27,083 to 75,554 in the VDE group while ranging from 0 to 254 in the Arkime group. **(C)** Confidence in answers. Whiskers are 95% confidence intervals.

The Kruskal-Wallis *H* test showed that the total number of correctly identified Red Team hosts targeting Blue Team systems was significantly different between the VDE group and the Arkime group (*p* < 0.001). Dunn's *post hoc* test showed that the VDE group identified significantly more correct Red Team hosts targeting Blue Team systems compared to the Arkime group (z = 3.58, *p*_bonf_ < 0.001). Figure 5A shows interval plots for the differences in correctly identified Red Team hosts targeting Blue Team systems.

The Kruskal-Wallis *H* test showed that the activity associated with the correctly identified Red Team hosts targeting Blue Team systems was significantly different between the VDE group and the Arkime group (*p* < 0.001). Dunn's *post hoc* test showed that the VDE group identified significantly more highly-active Red Team hosts targeting Blue Team systems compared to the Arkime group (z = 3.97, *p*_bonf_ < 0.001). Figure 5B shows interval plots for the differences in the traffic associated with correctly identified Red Team hosts targeting Blue Team systems.

One-Way ANOVA showed that confidence in having correctly identified Red Team hosts targeting Blue Team systems was significantly different between the VDE group and the Arkime group (*p* = 0.012). Tukey's *post hoc* test showed that the VDE group was significantly more confident in having correctly identified Red Team hosts targeting Blue Team systems compared to the Arkime group (MD = 27.45, *p*_bonf_ = 0.012). Figure 5C shows interval plots for the differences in how confident participants were in having identified the correct hosts.

#### 3.1.4. CSA 2: Identifying compromised BT segments

The Kruskal-Wallis *H* test showed that the number of identified Blue Team segments compromised by the Red Team was significantly different between the VDE group and the Arkime group (*p* = 0.003). Dunn's *post hoc* test showed that the VDE group identified significantly more Blue Team segments that were compromised by the Red Team compared to the Arkime group (z = 2.97, *p*_bonf_ = 0.001).

**Figure 6:**
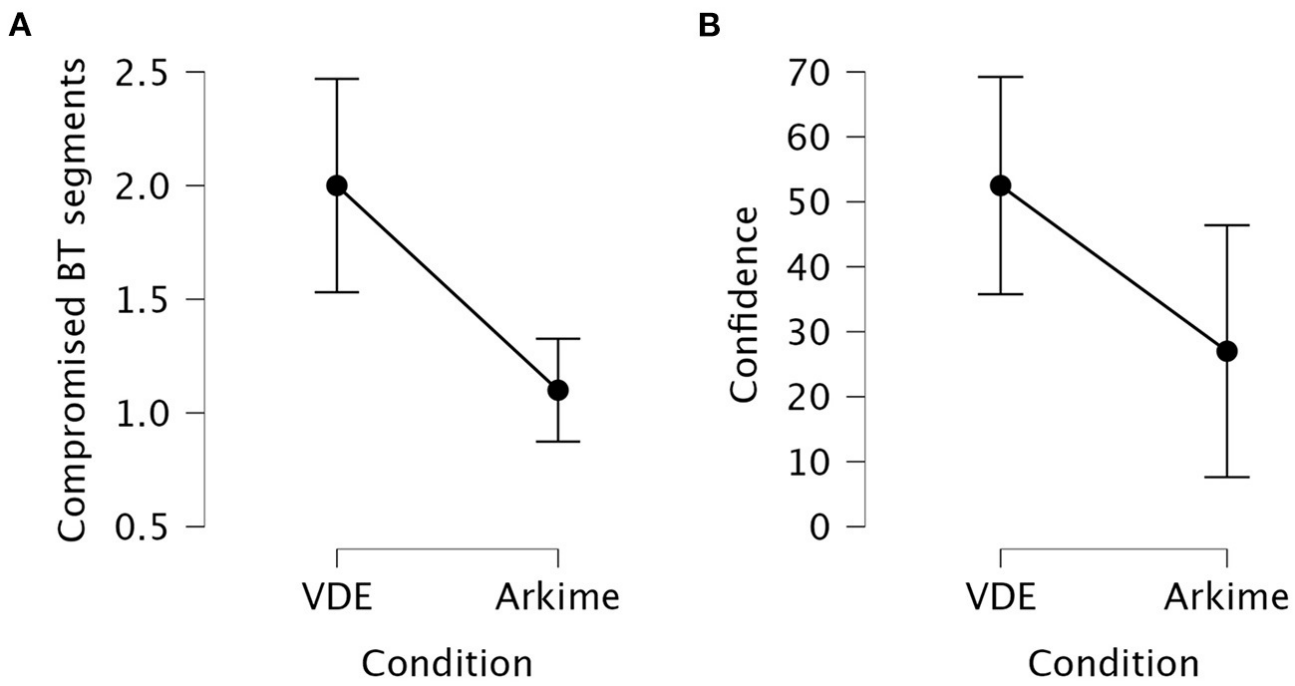
Interval plots for the differences in identifying compromised Blue Team systems. **(A)** Identified compromised Blue Team systems. **(B)** Confidence in having identified compromised Blue Team segments. Whiskers are 95% confidence intervals.

The Kruskal-Wallis *H* test showed that confidence in having correctly identified Blue Team segments compromised by the Red Team was significantly different between the VDE group and the Arkime group (*p* = 0.042). Dunn's *post hoc* test showed that the VDE group was significantly more confident in having correctly identified Blue Team segments compromised by the Red Team compared to the Arkime group (z = 2.03, *p*_bonf_ = 0.021). Figure 6 shows interval plots for differences between the VDE group and the Arkime group in having identified compromised Blue Team segments and confidence in having identified compromised Blue Team segments.

#### 3.1.5. CSA 3: Situational report

The Kruskal-Wallis *H* test showed that the accuracy score for the description of what type of activity they saw was significantly different between the VDE group and the Arkime group (*p* = 0.045). Dunn's *post hoc* test showed that the VDE group had a significantly higher accuracy score compared to the Arkime group (z = 2.00, *p*_bonf_ = 0.022).

The accuracy score for the description of type of incident it was (*p* = 0.098), the relevance score for the suggestion of actions that should be done (*p* = 0.320), and the thoroughness k-score (*p* = 0.081) were not significantly different between the groups.

One-Way ANOVA showed that confidence in the SITREP descriptions was significantly different between the VDE group and the Arkime group (*p* = 0.040). Tukey's *post hoc* test showed that the VDE group had a significantly higher confidence in their SITREP answers compared to the Arkime group (MD = 19.50, *p*_bonf_ = 0.040).

#### 3.1.6. CSA 4: Identifying BT hosts abused for RT lateral movements

The Kruskal-Wallis *H* test showed that neither the total number of Blue Team hosts abused for Red Team lateral movements (*p* = 0.188), the number of correctly identified Blue Team hosts abused for Red Team lateral movements (*p* = 0.055), nor the number of sessions associated with correctly identified Blue Team hosts abused for Red Team lateral movements (*p* = 0.473) were significantly different between the groups, although the difference in the number of correctly identified Blue Team hosts abused for Red Team lateral movements approached significance.

The Kruskal-Wallis *H* test showed that confidence in the answers was significantly different between the VDE group and the Arkime group (*p* = 0.010). Dunn's *post hoc* test showed that the VDE group had a significantly higher confidence in their answers compared to the Arkime group (z = 2.57, *p*_bonf_ = 0.005).

### 3.2. The effect of VDE on cyber team communication

#### 3.2.1. Self-reported communication demands

**Figure 7:**
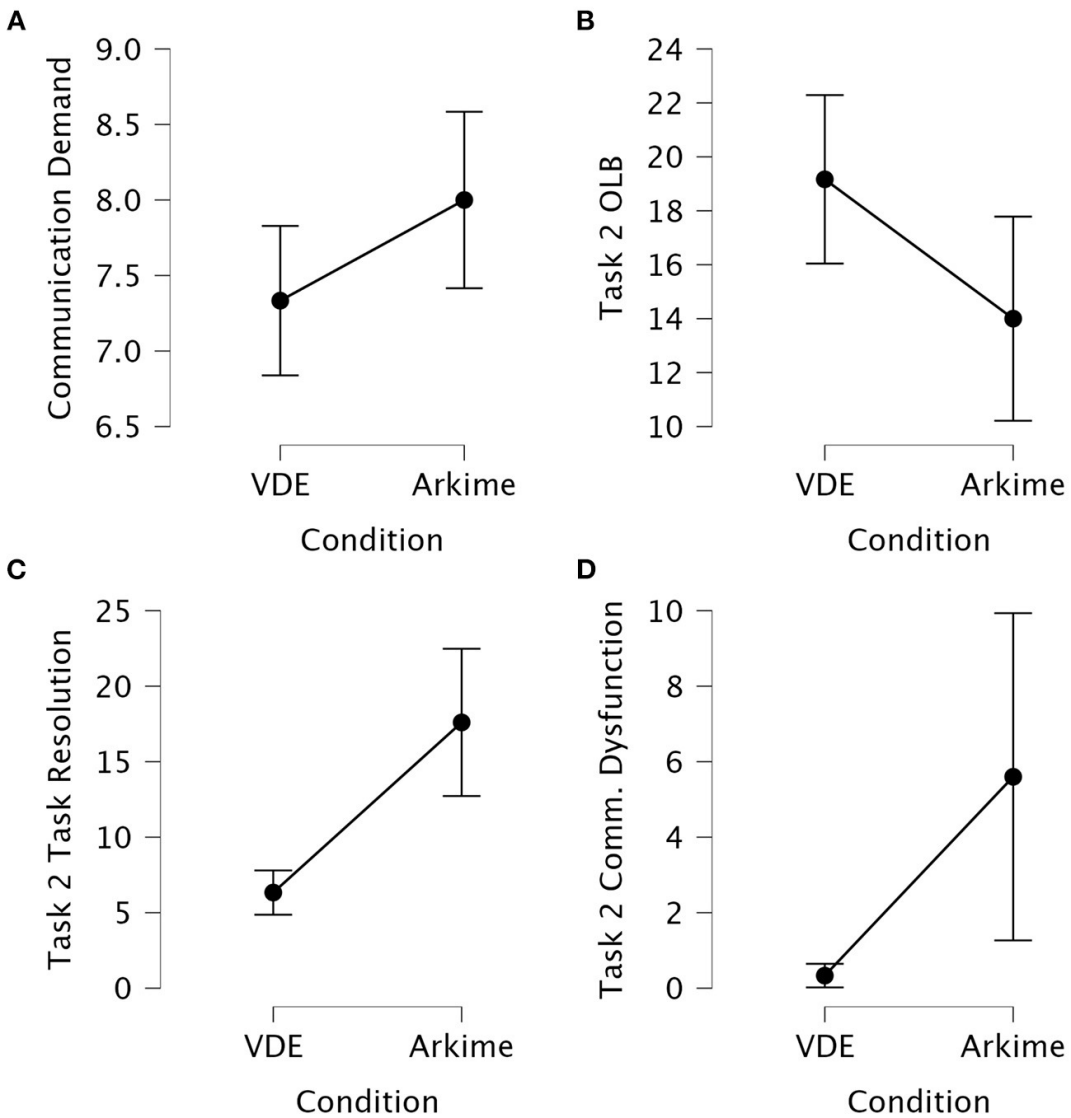
Interval plots for between-group differences in self-reported and observed communication variables. **(A)** Self-reported communication demands after part two of the experiment. **(B)** Observed task two OLB communication. **(C)** Observed task two task resolution communication. **(D)** Observed task two communication dysfunction. Whiskers are 95% confidence intervals.

The Kruskal-Wallis *H* test showed that the communication demands during part two of the experiment was significantly different between the VDE group and the Arkime group (*p* = 0.048). Dunn's *post hoc* test showed that the VDE group experienced significantly lower communication demands compared to the Arkime group (z = −1.98, *p*_bonf_ = 0.024). No other TWLQ measures were significantly different between the groups. Figure 7A shows interval plots displaying differences in part two communication demands between the groups.

#### 3.2.2. Observation of communication behaviors

Kruskal-Wallis *H* tests and One-Way ANOVAs were used to assess differences on the observed verbal communication scores between the VDE group and the Arkime group. Table 4 presents the result of the comparisons. Figures 7B–D shows interval plots for between-group differences in task two OLB communication, task two task resolution communication, and task two communication dysfunction.

**Table 4:** Comparison of observational scores between VDE and Arkime (*N* = 22).

		**Kruskal-Wallis test**			**Dunn's** ***post hoc***	
**Variables**	**M** ± **SD**	***H*** **(1)**	***p***	η^2^	**z**	*p*_bonf_
Task 1 OLB	10.45 ± 13.00	4.145	**0.042**	0.157	2.036	**0.021**
Task 1 perceptual shared mental models	9.00 ± 11.75	0.461	0.497	−0.026	-	-
Task 1 task resolution	6.36 ± 11.08	0.000	1.000	−0.50	-	-
Task 1 communication dysfunction	0.27 ± 0.63	0.000	1.000	−0.50	-	-
Task 1 time to finish (min)	11.72 ± 15.46	0.000	1.000	−0.50	-	-
Task 2 perceptual shared mental models	16.90 ± 5.54	0.018	0.894	−0.049	-	-
Task 2 task resolution	11.45 ± 7.46	15.968	**<0.001**	0.748	−3.996	**<0.001**
Task 2 communication dysfunction	2.72 ± 4.80	4.101	**0.043**	0.155	−2.025	**0.021**
Task 2 time to finish (min)	28.90 ± 9.12	13.013	**<0.001**	0.600	−3.607	**<0.001**
Task 3 OLB	6.09 ± 8.79	0.916	0.339	−0.004	-	-
Task 3 perceptual shared mental models	5.45 ± 7.96	1.162	0.281	0.008	-	-
Task 3 task resolution	3.00 ± 3.59	0.898	0.343	−0.005	-	-
Task 3 communication dysfunction	0.90 ± 1.54	1.825	0.177	0.041	-	-
Task 3 time to finish (min)	6.27 ± 7.09	0.299	0.585	−0.035	-	-
		**One-way ANOVA**			**Tukey's** ***post hoc***	
		***F*** **(1)**	***p***	ω^2^	**MD**	*p*_bonf_
Task 2 OLB	16.81 ± 5.62	5.625	**0.028**	0.174	5.167	**0.028**

η^2^, Effect size; *p*_bonf_, Bonferroni adjusted *p*-values; Bold, significant differences; OLB, Orient, Locate, Bridge; ω^2^, Effect size; MD, Mean difference.

The Kruskal-Wallis *H* test showed that the VDE group had significantly different task one OLB scores compared to the Arkime group (*p* = 0.042). Dunn's *post hoc* test showed that the VDE group performed significantly more OLB communications during task one compared to the Arkime group (z = 2.03, *p*_bonf_ = 0.021). No other comparisons from task one were significant.

The one-way ANOVA showed that the VDE group had significantly different task two OLB scores compared to the Arkime group (*p* = 0.028). Tukey's *post hoc* test showed that the VDE group performed significantly more OLB communications during task two compared to the Arkime group (MD = 5.16, *p*_bonf_ = 0.028).

The Kruskal-Wallis *H* test showed that the VDE group had significantly different task two task resolution scores compared to the Arkime group (*p* < 0.001). Dunn's *post hoc* test showed that the VDE group performed significantly less task resolution communications during task two compared to the Arkime group (z = −3.99, *p*_bonf_ < 0.001). The Kruskal-Wallis *H* test showed that the VDE group had significantly different task two communication dysfunction scores compared to the Arkime group (*p* = 0.043). Dunn's *post hoc* test showed that the VDE group had significantly less communication dysfunction during task two compared to the Arkime group (z = −2.02, *p*_bonf_ = 0.021). The Kruskal-Wallis *H* test showed that the VDE group had significantly different task two Time-to-finish scores compared to the Arkime group (*p* < 0.001). Dunn's *post hoc* test showed that the VDE group had significantly lower time-to-finish scores during task two compared to the Arkime group (z = −3.60, *p*_bonf_ < 0.001).

Perceptual shared mental models were not significantly different between the groups. No comparisons were significantly different between groups with respect to task three observational scores.

#### 3.2.3. Relationship between communication variables and CSA items

**Figure 8:**
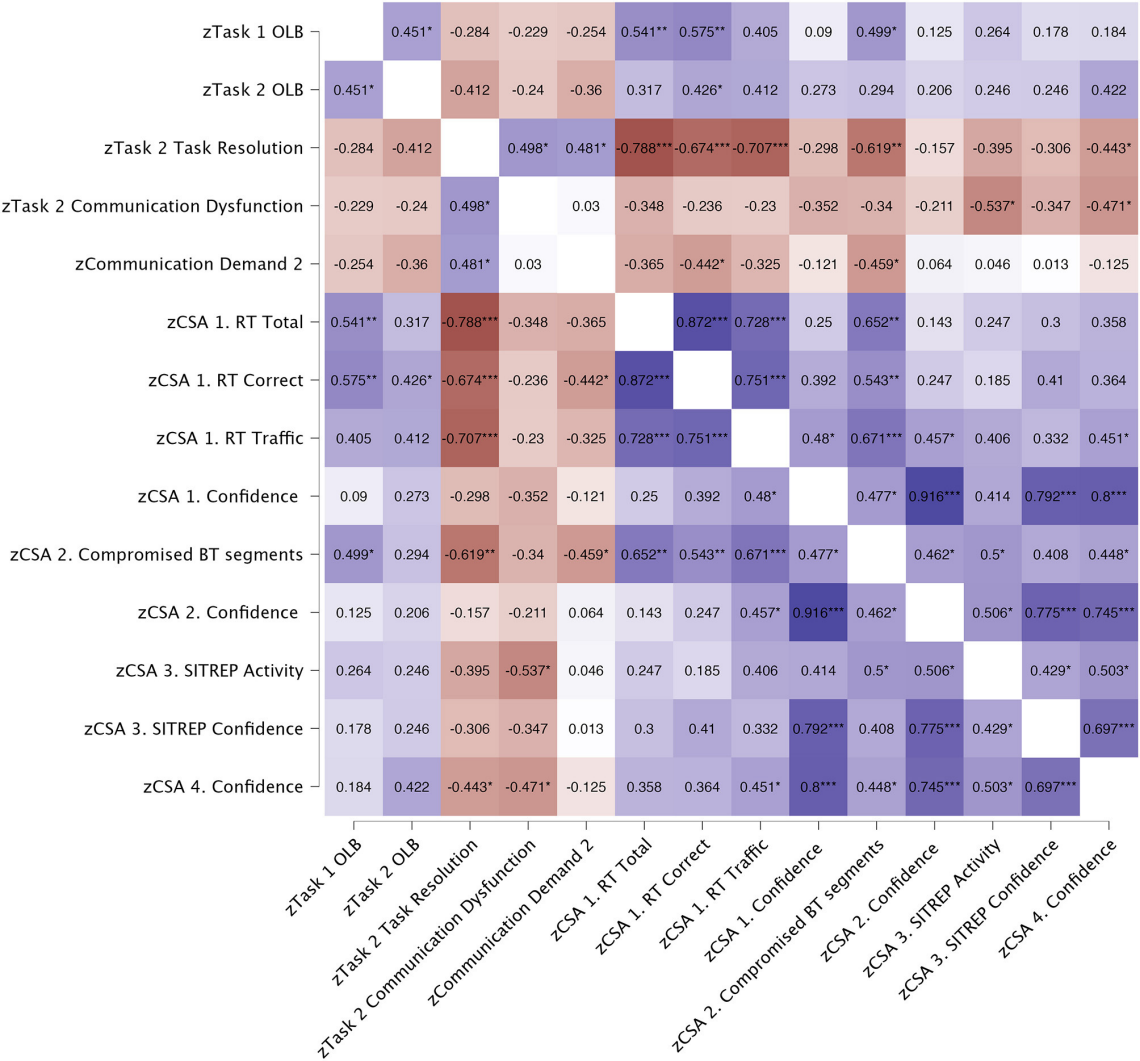
Heat map showing results from Spearman (ρ) correlations. All correlations are 2-tailed. Blue color indicates positive correlations. Red color indicates negative correlations. *p < 0.050, **p < 0.010, ***p < 0.001. OLB, Orient, Locate, Bridge (Knox et al., 2018); CSA, Cyber situational awareness; RT, Red team; BT, Blue team; SITREP, Situational report.

Spearman correlations were performed to assess the relationship between communication variables and CSA variables that were significantly different between the VDE group and the Arkime group. Figure 8 presents a heat map showing the results from the correlational analysis.

Task one OLB scores were significantly and positively correlated with task two OLB scores (*p* = 0.035), total number of identified Red Team hosts targeting Blue Team systems (*p* = 0.009), total number of correctly identified Red Team hosts targeting Blue Team systems (*p* = 0.005), and identifying compromised Blue Team segments (*p* = 0.018).

Task two OLB scores were significantly and positively correlated with total number of correctly identified Red Team hosts targeting Blue Team systems (*p* = 0.048).

Task two Task resolution scores were significantly and positively correlated with task two Communication dysfunction (*p* = 0.018), communication demands (*p* = 0.024), and negatively correlated with total number of identified Red Team hosts targeting Blue Team systems (*p* < 0.001), total number of correctly identified Red Team hosts targeting Blue Team systems (*p* < 0.001), total amount of traffic associated with correctly identified Red Team hosts targeting Blue Team systems (*p* < 0.001), identifying compromised Blue Team segments (*p* = 0.002), and confidence in having identified Blue Team hosts abused for Red Team lateral movements (*p* = 0.039).

Task two communication dysfunction scores were significantly and negatively correlated with the accuracy score for the description of what type of activity they saw (*p* = 0.010), and confidence in having identified Blue Team hosts abused for Red Team lateral movements (*p* = 0.027).

Part two communication demands were significantly and negatively correlated with total number of correctly identified Red Team hosts targeting Blue Team systems (*p* = 0.039), and identifying compromised Blue Team segments (*p* = 0.031).

No other correlations were significant.

Separate linear regressions were performed for significant correlations. Significant results are shown in Table 5. Task two task resolution was a significant negative predictor of total number of identified Red Team hosts targeting Blue Team systems (*p* < 0.001), total number of correctly identified Red Team hosts targeting Blue Team systems (*p* = 0.002), total amount of traffic associated with correctly identified Red Team hosts targeting Blue Team systems (*p* < 0.001), and identifying compromised Blue Team segments (*p* = 0.008). No other relationships were significant.

**Table 5:** Linear regressions (*N* = 22).

**Predictor**	**Dependent variable**	**β**	***p***	** $R_{\text{Adj}}^{2}$ **	***F* (1)**
Task two task resolution	RT hosts targeting BT systems total	−0.763	< 0.001	0.561	27.845
Task two task resolution	RT hosts targeting BT systems correct	−0.630	0.002	0.366	13.142
Task two task resolution	RT hosts targeting BT systems traffic	−0.665	< 0.001	0.415	15.889
Task two task resolution	Identifying compromised BT segments	−0.547	0.008	0.264	8.534
Task two communication dysfunction	SITREP—Describe the activity you saw	−0.524	0.012	0.238	7.553
Task two communication dysfunction	BT hosts abused for RT lateral movements confidence	−0.525	0.012	0.239	7.594
Communication demands	RT hosts targeting BT systems correct	−0.454	0.034	0.166	5.178

RT, Red team; BT, Blue team; SITREP, Situational report.

Task two communication dysfunction was a significant negative predictor of the accuracy score for the description of what type of activity they saw (*p* = 0.012), and confidence in having identified Blue Team hosts abused for Red Team lateral movements (*p* = 0.012).

Communication demands was a significant negative predictor of the total number of correctly identified Red Team hosts targeting Blue Team systems (*p* = 0.034). No other relationships were significant.

### 3.3. The effect of VDE on decision-making

All the participants except two (*n* = 20) picked the “Start incident response on selected hosts” option on the forced-choice decision-making task. Thus, there was no difference between the groups. The other two participants, both in the VDE condition but not in the same dyad, picked the “Cut off all connectivity from the friendly networks to outside” and the “Cut off connectivity to a selection of network segments” options.

## 4. Discussion

Cyber defense decision-making during CTSs is based on human communication aiming to establish a shared CSA between analyst-level and decision-making personnel (Knox et al., 2018). Communication for shared CSA is one of the main problems facing SOC team analysts (Knox et al., 2018; Agyepong et al., 2019; Ask et al., 2021a). Current visualization tools to support achieving a shared understanding of the CTS include 2D graphs and schematics of network topology. These visualization tools do not scale well with increasing complexity. Furthermore, SA level 3 appears to be the SA stage most dependent on human working memory (Gutzwiller and Clegg, 2013). The mammalian brain has developed an innate ability to understand time and space (Eichenbaum, 2014; Ray and Brecht, 2016; Berggaard et al., 2018). 3D representations of network topology may leverage automatic spatial sensory processes (Stackman et al., 2002; Angelaki and Cullen, 2008; Moser et al., 2008) that reduce load on working memory during communication. Thus, 3D visualizations may be more neuroergonomic than 2D representations by facilitating more efficient communication and shared situational understanding during CTSs, which could support decision-making (Kullman et al., 2019a). In this study, we compared how the representation of a network topology in 3D MR (Kullman et al., 2018, 2020) vs. 2D affected topology recognition, CSA, team communication and decision-making in a sample of cyber cadets.

In the first part of the experiment, the Arkime group performed better than the VDE group on the task where participants had to identify the correct depiction of the network topology among four 2D schematics. This finding was not surprising as the correct depiction was in the same format as the 2D schematic the Arkime group had used to familiarize themselves with the topology.

3D visualizations of network topology are expected to be neuroergonomic in the sense that they leverage innate neurocognitive processes that encode spatial information. Additionally, the VDE visualizes network data based on the mental model that operators have of the network they are defending (Kullman et al., 2020). While both being neuroergonomic and conserving connections and sessions between nodes, the topological layout as visualized in the VDE does not represent the actual reality of the network. This may be problematic if the 3D visualizations contribute to a false sense of confidence in one's understanding of the topology by virtue of being visually persuasive. For instance, previous studies on cyber cadets have shown that high self-confidence in combination with intuitive decision-making can have detrimental effects on performance when counterintuitive decisions are required (Lugo et al., 2016). Interestingly, while performing worse, the VDE group was also less confident in their answers on the topology recognition task. Thus, the 3D visualizations did not give a false sense of confidence with respect to topology recognition.

Awareness of adversarial behavior is suggested to be necessary for achieving CSA for cyber defense decision-making (Barford et al., 2009) although non-technical stakeholders may underestimate the importance of such information (Varga et al., 2018). This may have severe consequences for decision-making if analyst-level personnel and decision-makers have different mental models of the CTS and the network, especially if analyst-level personnel are not aware of this discrepancy during RCP communication (Ask et al., 2021a). Because the VDE allows for visualizing, thus sharing the mental models that the analyst have of the network topology (Kullman et al., 2018, 2020), this potential gap in information requirements (Varga et al., 2018) may be bridged more efficiently if adversarial behavior can be visualized during RCP sharing. While non-technical personnel were not included in the present study, the VDE group outperformed the Arkime group on all metrics when they were tasked to identify the top five Red Team hosts targeting Blue Team systems. This was true for correctly identifying Red Team hosts targeting Blue Team systems, but especially apparent for the traffic associated with the identified Red Team hosts where the differences in the session number associated with the identified connections differed in the tens of thousands. Moreover, the VDE group identified the connection with the highest amount of associated traffic while the Arkime group did not. Considering that the Arkime group could see the session number associated with the connections when hovering over the edge connecting the nodes while the VDE group had to go by edge brightness alone, this difference in performance is arguably the most salient of the experimental results.

Considering the role of working memory in SA (Gutzwiller and Clegg, 2013), it could be that using edge brightness as a cue for traffic provided an advantage over having access to actual session statistics due to complexity reduction freeing up cognitive resources. Albeit being allowed to write down their discoveries (e.g., host IP, session number), having the actual statistics available may result in deliberately or habitually engaging in analytical procedures that require the application of additional cognitive processes. This may include processes that tax attention allocation and working memory which could be detrimental to performance in a working environment that is already taxing on cognitive resources (Champion et al., 2012; Sawyer and Hancock, 2018). Alternatively, or additionally, it could be that having the network topology fixed in space and at a scale where participants could walk from node to node, facilitated a method of loci/memory palace-effect (Legge et al., 2012; Wagner et al., 2021), due to the spatial encoding of information (Stackman et al., 2002; Angelaki and Cullen, 2008; Moser et al., 2008). By using edge brightness as the singular attentional cue combined with a spatial layout, the VDE may have improved performance by allowing for increased ease of visuo-cognitive processing of the state of the network. But what if participants were tasked to find the bottom five Red Team hosts (e.g., rare or ambiguous signals) targeting Blue Team systems (with session number above zero)? For instance, would edge brightness then be distracting, or would the differences in performance remain? This question should be addressed in future studies.

Interestingly, and without knowing that they had outperformed participants in the Arkime group, some of the participants in the VDE condition expressed that they would have liked to have session number available for inquiry. This may further suggest that taxing habitual or procedural (e.g., trained) cognitive processes could have contributed to performance differences between the groups. In a realistic scenario, however, the VDE would not be used to replace packet capture software or any investigative tools. Instead, the SOC analysts would have all their usual tools available to them, while the VDE would be an additional tool that analysts could use to interact with network data according to their information processing needs (Kullman and Engel, 2022a,b). If analysts would prefer to inquire about session statistics, they could either probe for that through common means or incorporate it in VDE. This, in turn, serves to deepen their understanding of the cyber environment they are operating within on their own terms, either for themselves or when communicating with team analysts, decision-makers, or stakeholders (Kullman et al., 2019a).

Awareness of the impact of an attack is also suggested to be necessary for achieving CSA for good cyber defense decision-making (Barford et al., 2009). In the present study, the VDE group identified more Blue Team segments that were compromised by the Red Team than the Arkime group. Given the level of uncertainty that is inherent to the cyber domain (Jøsok et al., 2016), this difference in impact awareness may be advantageous when attempting to reduce the level of experienced uncertainty both when attempting to understand the situation but also perhaps when evaluating the trustworthiness of CSA information, especially for non-technical personnel. The latter is also suggested to be important for achieving CSA for cyber defense decision-making (Barford et al., 2009).

To assess the potential effect of VDE on RCP communication, we asked participants to provide a short situational report based on three open-ended questions which were later used to generate three scores based on accuracy and relevance. In line with Barford et al. (2009), the questions were aimed at measuring (a) awareness of the current situation by describing the activity they saw, (b) what caused it by describing what type of incident it was, and (c) how the situation may evolve by suggesting which actions should be taken. A k-score was generated based on the overall thoroughness of the situational report. Although the VDE group scored higher than the Arkime group on all four measures, only the activity description score was significantly different between the groups.

In the present study, the VDE group identified more Blue Team hosts that were abused for Red Team lateral movements. However, this was not significantly different between the groups (although the number of correctly identified abused Blue Team hosts approached significance). Considering the difference in performance on task two, the lack of difference in performance on task three could be due to the time limit that the participants had to work under. It could also be due to the limited sample size. This will have to be addressed in future studies.

During the second part of the experiment, the VDE group was more confident in their answers than the Arkime group on all CSA measures. This should be considered in light of the fact that the VDE group performed significantly better than the Arkime group on several of the performance outcomes while having higher scores on all performance outcomes (although not all were significantly different). The outcome measures for the fourth CSA question (the question relating to task three) was the only measure where not one of the scores were significantly different between the groups. When also considering the lower confidence scores when the VDE group actually performed worse than the Arkime group, it could indicate that these performance estimations are well-founded. A second interpretation could be that the cyber cadets have good metacognitive accuracy irrespective of the conditions they were assigned to. Previous studies on cyber cadets have indicated that they are similar in their cognitive profiles (Lugo and Sütterlin, 2018), and that cyber cadets with higher metacognitive accuracy have better CSA, while overconfident cyber cadets have worse CSA (Ask et al., 2023). Assessing the metacognitive accuracy of the participants with respect to performance outcomes will be addressed in the study examining the cognitive measures that were taken during the experiment.

It is important to restate here that the VDE is not a tool for conducting forensic analyses *per se*. It is a neuroergonomic tool for visualizing network topology in accordance with the analyst's mental model of the network (Kullman and Engel, 2022a,b). This allows the analyst to not have to spend working memory on mentally maintaining or navigating the representation of their mental models when they are seeking to understand a CTS. Because individuals collaborating in VDE will have the same spatial mental model of the network (Kullman and Engel, 2022a,b), less mental effort may be required to ground communication, thus making knowledge transfer more efficient. While the experimental tasks and preliminary nature of the present study does not capture traditional SOC activities with sufficient realism, it still goes some way in capturing how the VDE influences communication processes when individuals are collaborating to establish CSA.

During the second part of the experiment, participants in the VDE condition experienced a lower communication demand compared to participants in the Arkime condition, suggesting that the VDE improves communication efficiency. Thus, when considering that communication inefficiencies are one of the biggest but least researched problems facing SOC team analysts (Agyepong et al., 2019; Ask et al., 2021a), this finding may indicate that the VDE could aid in solving some of those communication problems.

Previous studies have indicated that task-related communication is different between poor and well performing cyber teams during CDXs (Jariwala et al., 2012; Ask et al., 2023) but that expert cyber analysts communicate less than novice cyber analysts (Buchler et al., 2016; Lugo et al., 2017). This could indicate that experts communicate more effectively (e.g., are better at OLB processes; Knox et al., 2018) and more readily achieve a shared mental model of the tasks they are solving and of the cyber threat situation (Ask et al., 2023). A recent review found that there was a lack of studies characterizing the communication in cyber defense settings such as the purpose of communication and the type of communication (Ask et al., 2021a). In the present study, we noted the frequency of dyadic verbal communication as they related to OLB processes, task resolution, achieving a shared perceptual mental model, and communication dysfunction. We found that the VDE group performed significantly more OLB communication (which are aimed at achieving a shared understanding of a situation; Knox et al., 2018) during task one and task two, while the Arkime group performed significantly more task resolution communications and had more communication dysfunctions during task two. In our regression analysis, both observed and self-reported communication variables that were scored higher in the Arkime group compared to the VDE group were negative predictors of CSA scores. This could indicate that the VDE facilitates more efficient cyber team communication and should be assessed further in future studies. The possibility for using VDE in remote dyadic cooperation should also be assessed in future studies to assess whether these potential effects are present when body language cues are not available to the participants.

In the present study, almost all participants picked the same decision regardless of assigned condition or individual performance. This is likely due to cohort effects such as training but could also potentially be due to the specific cognitive profiles that the cyber engineering profession selects for Lugo and Sütterlin (2018). This could explain why the relevance score for the actions suggested in the situation report were not different between the groups. Future studies should include a more diverse sample to avoid potential confounding influences on the effect of VDE on decision-making. Because the VDE visualizations are established through an interview with the user of the visualizations (the analysts; Kullman et al., 2018, 2020), the 3D layout of the network topology in VDE is generated through user-centric cooperative-design principles. Due to the participants not being familiar with the network they were working with in the current experiment, the 3D layout was predefined. Usually, a cyber analyst will know the network they are operating within, thus, there is always a possibility that the unfamiliarity of the network made participants choose “safer” and similar decision-making options.

### 4.1. Limitations and future perspectives

The present study has a few limitations. The VDE group had higher scores on all performance measures and lower scores on all team workload measures during the second part of the experiment, although not all of these were significantly different. It is hard to say whether differences would have reached significance with a larger sample size. Considering this possibility, the experiment should be repeated in a larger sample.

With most behavioral experiments, there is a question of whether the experimental design produces results that can be generalized to a real-world setting. Due to being high stakes and unfolding in a complex working environment, defensive cyber operations can be stressful and often entail being exposed to a number of distractors (e.g., security alerts that are false positives) that may degrade performance over time (Champion et al., 2012; Sawyer and Hancock, 2018). Future studies should therefore include more distractors to ensure that results have high ecological value. This could include explicating time limits on all tasks, or exposing participants to periodic security alerts and increasing indicators of compromise (scenario injections). This in turn would allow the assessment of how taxing different senses and cognitive systems affect VDE vs. Arkime usability for CSA generation, team communication, and decision-making. Furthermore, applying the VDE in a setting that captures SOC tasks with more realism, including analyst-to-decision-maker communication will be necessary to fully validate the potential usability of the VDE for achieving a shared CSA.

While the overall performance of the HoloLens 2 was good, there were some instances where the HoloLens 2 headsets overheated which negatively affected application's stability and forced a few minute-long breaks while the headset was being replaced. Wearing a battery pack that provided the HoloLens 2 device with additional power appeared to solve the problem but the form factor of the battery pack and absence of dedicated gear (the participants kept the battery in their pocket) made it a somewhat awkward experience. This should be addressed in future research to ensure a more seamless experience that works under various conditions.

## 5. Conclusions

In the present study, a collaborative, 3D mixed reality representation of a network topology and network attack provided better CSA compared to using paper-based, 2D topology schematics and graph representation in the packet capture software Arkime. The most apparent difference was in the detection of the top five Red Team hosts targeting Blue Team systems. The traffic associated with the identified Red Team hosts in the mixed reality condition differed in the tens of thousands. This is remarkable, as participants in the mixed reality condition could only use edge brightness as a cue for traffic while participants in the Arkime condition could see the actual session number statistics. Observed and self-reported communication was better for dyads in the VDE condition and was associated with their CSA. This may suggest that the VDE has neuroergonomic benefits when SOC team analysts need to communicate for shared CSA. Although participants in the mixed reality condition had higher CSA, we were not able to measure its effect on decision-making. This could be due to cohort effects such as training or the modest sample size. Finally, the experimental tasks and preliminary nature of the study does not reflect SOC tasks with sufficient realism. Thus, to truly assess the potential effects of VDE on communication for shared CSA, the study should be repeated in a naturalistic setting with a larger and more diverse sample.

## Data availability statement

The datasets presented in this article are not readily available because access to raw and processed data is restricted in accordance with agreement between the researchers and the Norwegian Defense University College, Cyber Academy (NDCA).

## Ethics statement

Ethical review and approval was not required for the study on human participants in accordance with the local legislation and institutional requirements. The patients/participants provided their written informed consent to participate in this study.

## Author contributions

TA: experimental design, data collection, statistical analysis, and writing the original draft, review, and editing. KK: development of the data visualization application, experimental design, and writing, review, and editing of the original draft. SS: experimental design, review, and editing of original draft. BK: experimental design, data collection, review, and editing of original draft. DE: writing, review, and editing. RL: experimental design, data collection, statistical analysis, and writing, review, and editing of original draft. All authors approved the final draft of the manuscript.

## Footnotes

^1^RCN #302941. Project website: https://www.hiof.no/hvo/vlo/english/research/projects/acdicom/.
